# Supplementary material for: A pair of non-Mendelian genes at the Ga2 locus confer unilateral cross-incompatibility in maize
Source: Nat Commun. 2022 Apr 14;13:1993. doi: 10.1038/s41467-022-29729-z (PMC9010485; doi:10.1038/s41467-022-29729-z)
Supplement: Supplementary file 1 — Supplementary information [file 41467_2022_29729_MOESM1_ESM.pdf]

A pair of non-Mendelian genes at the *Ga2* locus confer unilateral cross-incompatibility in maize

Chen *et al*.

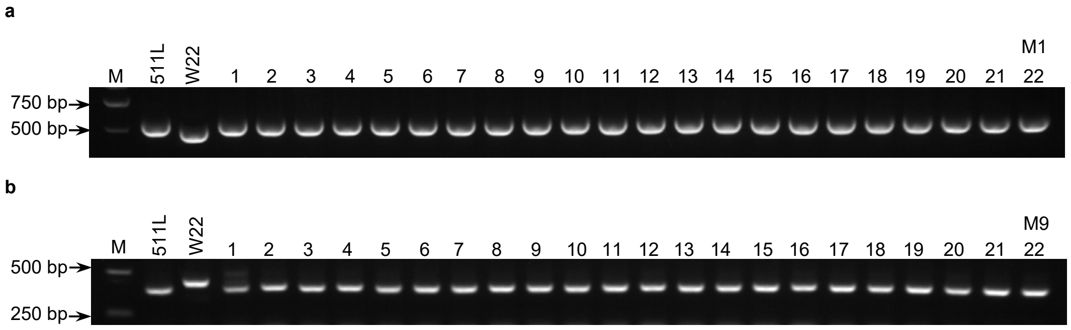

Supplementary Fig. 1 Genetic analysis of the male determinant.

a and **b** Genotyping of the 511L (♀)×(W22×511L) (♂) BC_1F_1 population using DNA markers M1 and M9 between W22 and 511L flanking the *Ga2* locus. Source data are provided as a Source Data file.

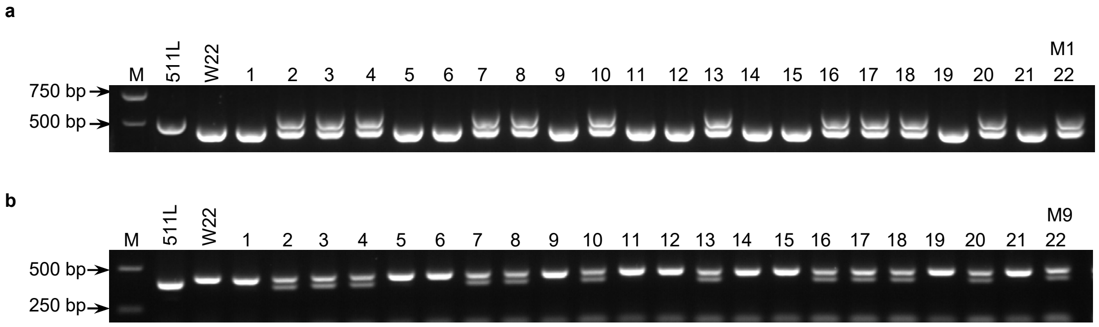

Supplementary Fig. 2 Genetic analysis of the female determinant.

a and **b** Genotyping of the (W22×511L) (♀)×W22 (♂) BC_1F_1 population using DNA markers M1 and M9 between W22 and 511L flanking the *Ga2* locus. Source data are provided as a Source Data file.

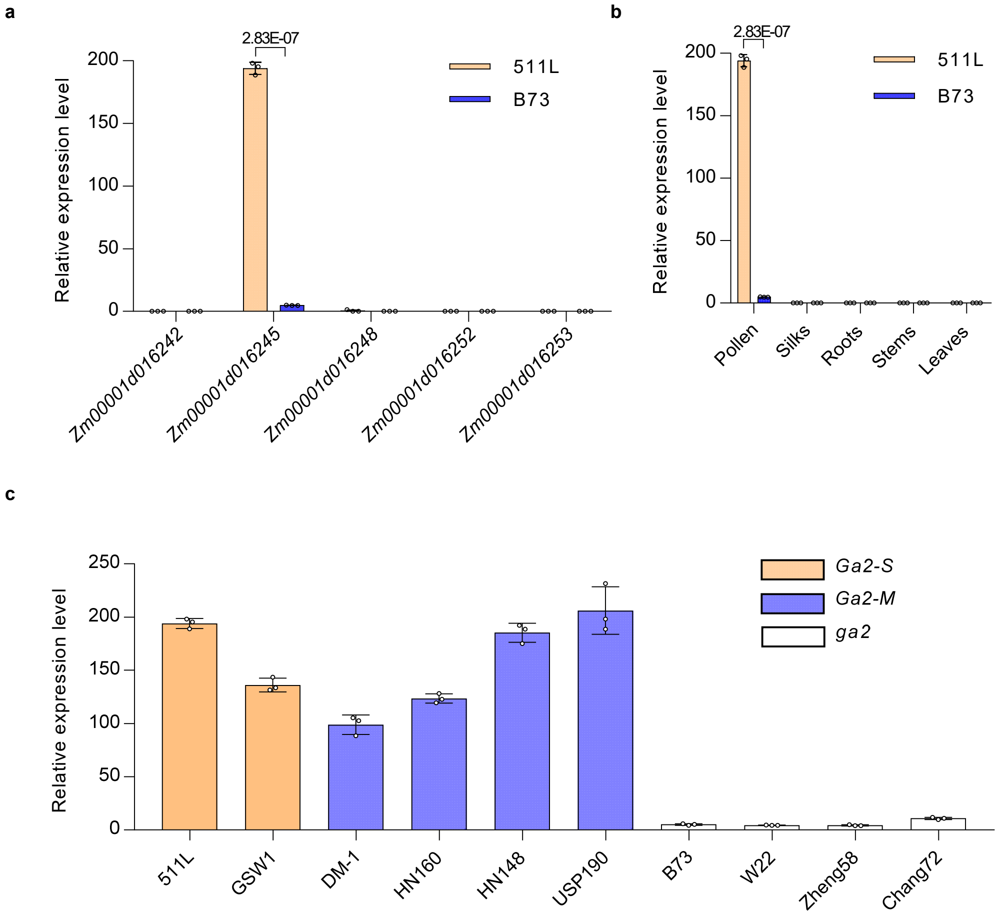

Supplementary Fig. 3 *Zm00001d016245* is specifically expressed in *Ga2* pollen.

a Expression analysis of five annotated genes within the mapping region in pollens of 511L and B73.

b Spatial expression analysis of *Zm00001d016245* in 511L and B73.

c Expression analysis of *Zm00001d016245* in *Ga2-S*, *Ga2-M* and *ga2* pollen.

ZmGAPDH was used as an internal control. Expression means and Standard Errors were from 3 biological replicates (unpaired two-tailed Student's *t*-test). The *P* values are indicated in the graphs. Source data are provided as a Source Data file.

```
511L ATGGAGAGCAGGAGGAAGAATCAACAGAAGACGAGCTACATTGTAATTTCCATCCAGCTCCTAGCGGCCCTCCTACTCTTGGAGCTTGTG 90
B73  ATGGAGAGCAGGAGGAAGAATCAACAGAAGACGAGCTACATTGTAATTTCCATCCAGCTCCTAGCGGCCCTCCTACTCTTGGAGCTTGTG 90
     ******************************************************************************************

511L TCGCTAGTGCCGGGGGCGTCATGCGAGGAGTTGCCCTTGGATTTTTGGGTGAGTGCACTACGGGGGGTCGGGGCAAAGGACGTTTCAACA 180
B73  TCGCTAGTGCCGGGGGCGTCATGCGAGGAGTTGCCCTTGGATTTTTGGGTGAGTGCACTACGGGGGGTCGGGGCAAAGGACGTTTCAACA 180
     ******************************************************************************************

511L GCCGATGGATGCACCAAGAAGAATAAGGACACCGTGCTCTGCTCTGCCCAGGCTAACACGGTAACCAATTTCATCAACCCTACCAACTCT 270
B73  GCCGATGGATGCACCAAGAAGAATAAGGACACCATGCTCTGCTCTGCCCAGGGTAACACAGTAACCAATTTCATCAACCCTACCAACTCT 270
     ********************************* ****************** ****** ******************************

511L GAGGAACAAGGCTATAGGACCATCGGGGAGTCCATCGCTAACATCCCTGATGATAGCACCAAACGGTACATCCTCATCCTCAGCGGTGGC 360
B73  GAGGAACAAGGCTATAGGACCATCGGG-AGTCCATCGCTAACATCCCTGATGATAGCACCAAACGGTACATCCTCATCCTCAGCGGTGGC 359
     *************************** **************************************************************

511L ACCGTGTACCGAGAGAAGGTATTGGTGAGCAAAAGCAAGCCATTCGTCACCATAAGATCAGATGACCCCATCAACCCTGCCATCATTGTG 450
B73  ACCGTGTACCGAGAGAAGGTATTGGTGAGCAAAAGCAAGCCATTCGTCACCATAAGATCAGATGACCCCATCAACCCTGCCATCATTGTG 449
     *************************   **************************************************************

511L TGGAACGACACTGCCGCCACCCTGGGGAAGGATAGCAAGCCCCTTGGAGTAGATGGTAGTAGCACCATGACCGTAGAGTCCGACTACTTC 540
B73  TGGAACGACACTGCCGCCACCCTGGGGAAGGATAGCAAGCCCCTTGGAGTAGATGGTAGTAGCACCATGACCGTAGAGTCCGACTACTTC 539
     ******************************************************************************************

511L ATTGCCTATGGTGTCGTCTTTAGGAATGATGCTGCAGCAGCAGCGAAGAAGAAGAAGGCAGAAGGCGAGGCGCCAGCGTTGCGGGTGCTA 630
B73  ATTGCCTATGGTGTCGTCTTTAGGAATGATGCTGCAGCAGCAGCGAAGAAGAAGAAGGCAGAAGGCGAGGCGCCAGCGTTGCGGGTGCTA 629
     ******************************************************************************************

511L GGAACAAAGGCAACCTTCTACAACTGCACAATTGAAGGTGGACAAGGCGCCCTGTATGACCAGATGGGGCTGCACTACTTCAAGTCCTGC 720
B73  GGAACAAAGGCAACCTTCTACAACTGCACAATTGAAGGTGGACAAGGCGCCCTGTATGACCAGATGGGGCTGCACTACTTCAAGTCCTGC 719
     ******************************************************************************************

511L ACCATCAGGGGAACCATCGACTTCATCTTTGGCTCTGCCAAGTCCTTTTACGAGGACTGCACCATTGTTTCCGTGAACAACATGGAGGAG 810
B73  ACCATCAGGGGCACCATCGACTTCATCTTTGGCTCTGCCAAGTCCTTTTATGAGGACTGCACCATTGTTTCCGTGAACAACATGGAGGAG 809
     *********** ************************************** ***************************************

511L ATCATGACCTTGCCTGTGGCACCACCTCAACTTGACATTCACGACAATCCAATCAAAGTTGCCCCAGGGGAGGGCGGCTTCTCCTTCAAG 900
B73  ATCATGACCTTGCCTGTGGCACCACCTCAACTTGACATTCACGACAATCCAATCAAAGTTGCCCCAGGGAAGGGAGGCTTCTCCTTCAAG 899
     ********************************************************************* **** ***************

511L ACATGTACCATCACTGGGGAAGGGCAACAAATCTTCCTCGGAAGGATGGGCACGCCTTCCATCTACTCCTACACCCAGATTGCTAAGGAG 990
B73  ACATGTACCATCACCGGGGAAGGGCAACAAATCTTCCTCGGAAGGATGGGCACGCCTTCCATCTACTCCTACACCCAGATTGCTAAGGAG 989
     ************** ***************************************************************************

511L GTTGTGCCCATAATCTACGACAAAGGGAACATCTTCATGCCCAGTAATATGTATGCCGACCCATGCATGCAACAAAATCTCTCTCTCTCT 1,080
B73  GTTGTGCCCATAATCTACGACAAAGGGAACATCTTCATGCCCAGTAATATGTATGCCGACCCATGCATGCAGCAAAATCTCTCTCTCTCT 1,079
     *********************************************************************** ******************

511L CTTTATATATATATATATACACTTGTTATCTCATATCTATATCTATGCAGGACTGGTAGACGCTGTGCCACTTTCAAGTGCTATGGACCT 1,170
B73  CTTTATATATATATA----CACTTGTTATCTCATATCTATATCTATGCAGGACTGGTAGACGCTGTGCCACTTTCAAGTGCTATGGACCT 1,161
     ***************    ***********************************************************************

511L GGGTTAGAGAAAATATGGCACGTCAAACTTAGATACGCTGAAGCCATATACTTTCTTGGGACAGATTTTATCAACGGAGATTCATGGATC 1,260
B73  GGGTTAGAGAAAATATGGCACGTCAAACTTAGATACGCTGAAGCCATATACTTTCTTGGGACAGATTTTATCAACGGAGATTCATGGATC 1,251
     ******************************************************************************************

511L CTGTCCATACCACCTACTGATGCTGAAACATTGCTATCAGTTTGA 1,305
B73  CTGTCCATACCACCTACTGATGCTGAAACATTGCTATCAGTTTGA 1,300
     *********************************************
```

Supplementary Fig. 4 Alignment of *ZmGa2P* genomic sequence between 511L and B73.

The two exons in 511L are indicated by underlines. * indicates the identical nucleotides, and dash lines represent insertions or deletions. The premature stop codon of *ZmGa2P* in B73 is indicated in red.

```
511L MESRRKNQQKTSYIVISIQLLAALLLLELVSLVPGASCEELPLDFWVSALRGVGAKDVSTADGCTKKNKDTVLCSAQANTVTNFINPTNS 90
B73  MESRRKNQQKTSYIVISIQLLAALLLLELVSLVPGASCEELPLDFWVSALRGVGAKDVSTADGCTKKNKDTMLCSAQGNTVTNFINPTNS 90
     *********************************************************************** ***** ************

511L EEQGYRTIGESIANIPDDSTKRYILILSGGTVYREKVLVSKSKPFVTIRSDDPINPAIIVWNDTAATLGKDSKPLGVDGSSTMTVESDYF 180
B73  EEQGYRTIGSPSLTSLMIAPNGTSSSSAVAPCTERRYW---------------------------------------------------- 128
     *********

511L IAYGVVFRNDAAAAAKKKKAEGEAPALRVLGTKATFYNCTIEGGQGALYDQMGLHYFKSCTIRGTIDFIFGSAKSFYEDCTIVSVNNMEE 270
B73  ------------------------------------------------------------------------------------------ 128

511L IMTLPVAPPQLDIHDNPIKVAPGEGGFSFKTCTITGEGQQIFLGRMGTPSIYSYTQIAKEVVPIIYDKGNIFMPSNMTGRRCATFKCYGP 360
B73  ------------------------------------------------------------------------------------------ 128

511L GLEKIWHVKLRYAEAIYFLGTDFINGDSWILSIPPTDAETLLSV 404
B73  -------------------------------------------- 128
```

Supplementary Fig. 5 Alignment of *ZmGa2P* predicted protein sequence between 511L and B73.

* indicates the identical amino acids.

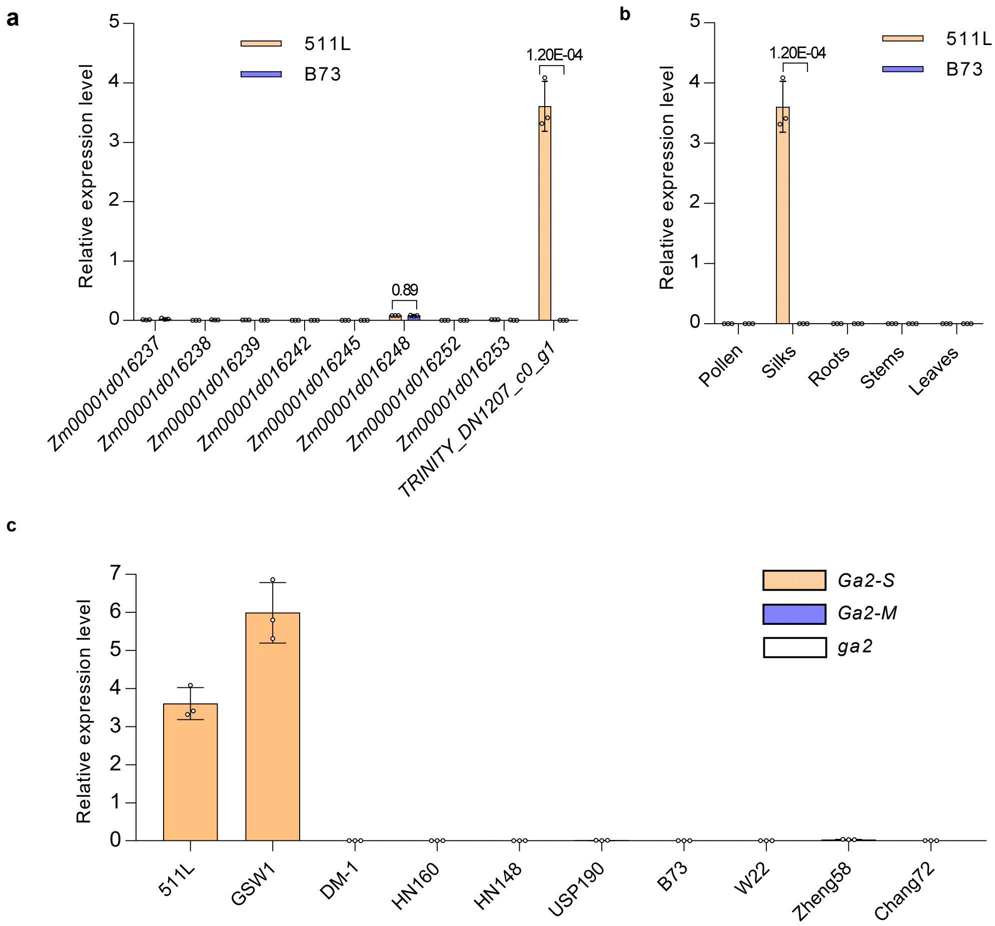

Supplementary Fig. 6 *TRINITY_DN1207_c0_g1* is specifically expressed in *Ga2-S* silks.

a Expression analysis of eight annotated genes within the mapping region in silks of 511L and B73.

b Spatial expression analysis of *TRINITY_DN1207_c0_g1* in 511L and B73.

c Expression analysis of *TRINITY_DN1207_c0_g1* transcript in *Ga2-S*, *Ga2-M* and *ga2* silks.

ZmGAPDH was used as an internal control. Expression means and Standard Errors were from 3 biological replicates (unpaired two-tailed Student's *t*-test). The *P* values are indicated in the graphs. Source data are provided as a Source Data file.

```
511L ATGGCAGCAGTTGGCAGATCGTCGCTGTCGCTCGTGCTGGTGATGGCGGCGGTGATCATCTCCGGCGCCGGTGGCGAGCGGGTGCCTCCT 90
B73  ATGGCAGCAGTTGGCAGATCGTCGCTGTCGCTCGTGCTGGTGATGGCGGCGGTGATCATCTCTGGCGCCGGTAGCGAGCGGGTGCCTCCT 90
     ************************************************************** ******** ******************

511L GCCGCAAA-GGAGCCCTGGCCGCCGGCTCCGCATGCTGTGGTCGTACAGCAGGCGCGGCTGGAGGAGTGCCACCGAGTGGCTCCGGCGTG 179
B73  GCCGCAAAAGGAGCCCTGGCCGCCGGCTCCGCATGCTGTGGTCGTACAGCAGGCGCGGCTGGAGGAGTGCCGCCGAGTGGCTCCGGCGTG 180
     *********************************************************************** ******************

511L CTACACCAGCATCGGACAAGCTCTGGCTGAAGCTGGCAAGCTTGTCCCCAAGGTGAAGCACCGTTTTGTTGTACTCATCAAGACCGGAGA 269
B73  CTACACCAGCATCGGACAAGCTCTGGCTGAAGCTGGCAAGCTTGTCCCCAAGGTGAAGCACCGTTTTGTTGTACTCATCAAGACCGGAGA 270
     ***************************   ************************************************************

511L GTACATAGAACAGGTGAACATCACGAGACGGAACGTCGTCCTGCTCGGCGAGGGCAGAGGCAACACAGTAATTTCTGGCAACCTAAGCAA 359
B73  GTACATAGAACAGGTGAACATCACGAGACGGAACGTCGTCCTGCTCGGCGAGGGCAGAGGCAACACAGTAATTTCTGGCAACCTAAGCAA 360
     ******************************************************************************************

511L CCTAACGGGGACGGCGATGGTGATGACGGCCACCGTGAGTAAGTGCTCCGATCGACGATCGACGTCGTCGTCTTCCGATCCGATGATTTG 449
B73  CCTAACGGGGACGGCGATGGTGATGACGGCCACCGTGAGTAAGTGCTCCGATCGACGATCGGCGTCGTCGTCTTCCGATCCGATGATTTG 450
     ************************************************************* ****************************

511L CTAGCTAACTAGATCTGTCATATGCATCACACACAGGCATGCATATATGCGATAGTAACTAACATTGACATTTTATTTTATTATGCATGC 539
B73  CTAGCTAACTAGATCTGTCATATGCATCACACACAGACATGCATATATGTGATAGTAACTAACATTGACATTTTATTTTATTATGCATGC 540
     ************************************ ************ ****************************************

511L ATGCAGATGTTGTAGCCGACGGCTTTTTGGCCCAGAACCTGACGATCCGTAACGAGGCCGGGCCGAAAGGAAGGCAGGCGGTGGCTTTGA 629
B73  ATGCAGATGTTGTAGCCGACGGCTTTTTGGCCCAGAACCTGACGATCCGTAACAAGGCCGGGCCGAAAGGAAGGCAGGCGGTGGCTTTGA 630
     ***************************************************** ************************************

511L GGTCAAACTCCAACCGGACCGTCGTATTTGGCTGTGCTATAGAAGGCTTCGAAGATAGCCTGTACGCCGAAAATGGAGTCCAGGTGTACC 719
B73  GGTCAAACTCCAACCGGACCGTCGTATTTGGCTGCGCTATAGAAGGCTTCGAAGATAGCCTGTACGCCGAAAATGGAGTCCAGGTGTACC 720
     ********************************** *******************************************************

511L TGGAGACGGACATATACGGGACCGTGGATTTTATATTCGGAAACGCGAAGGCGGTCTTCCAGCGTTGCCGCATCCTGGTGCGGCGGCCCA 809
B73  TGGAGACGGACATATACGGGACCGTGGATTTTATATTCGGAAACGCGAAGGCGGTCTTCCAGCGTTGCCGCATCCGGGTGCGGCGGCCCA 810
     ************************************************************************** ***************

511L TCCCCGGCAAGCACAACGTGGTGACGGCGCAGGGCTGCGACAACAATATGTACGAGAACTCCGGCTTCGTCTTCCACCGGTGCAGCGTCG 899
B73  TCCCCGGCAAGCACAACGTGGTGACGGCGCAGGGCTGCGACAACAATATGTACGAGAACTCCGGCTTCGTCTTCCACCGGTGCAGCGTCG 900
     ******************************************************************************************

511L AAGCTGACCCGAATCCATGGCCGGTGGGCCAGAACCTGACCGGCGTGGAAACATTTCTGGGCCGGCCGTACAGGAAGTACTCCCACGTCA 989
B73  AAGCTGACCCGAATCCATGGCCGGTGGGCCAGAACCTGACCGGCGTGGAAACATTTCTGGGCCGGCCGTACAGGAAGTACTCCCACGTCA 990
     ******************************************************************************************

511L TTTTCATGGAGTGCCAACTCAGCGACGTCGTGAGCGCCGCCGGCTGGGTGGCGTGGGACAGGGCACACGTCATCAATGATACCACGAAGA 1,079
B73  TTTTCATGGAGTGCCAACTCAGCGACGTCGTGAG-------------------------------------------------------- 1,024
     **********************************

511L GCGTGAGATACATGGAGTATGCCAACACAGGTCCCGGCGCCGACACCACCCACCGCGTCGACTGGGAGGGCGTCCATGTTCTCCACGACC 1,169
B73  ------------------------------------------------------------------------------------------ 1,024

511L CCGCCCAGGTAGCCAAATACACGATAGATGCCTTCATATCGGGTAAGGAGTGGATTCCCCATCAGATCCCGTATGACCATGAGGTCCCCA 1,259
B73  ------------------------------------------------------------------------------------------

511L GCGGGCGCGGCGCCATCATCGTCAACTAG 1,288
B73  ----------------------------- 1,024
```

Supplementary Fig. 7 Alignment of *ZmGa2F* genomic sequence between 511L and B73*.* The two exons in 511L are indicated by underlines. * indicates the identical nucleotides, and dash lines represent insertions or deletions. The premature stop codon of *ZmGa2F* in B73 is indicated in red.

```
511L MAAVGRSSLSLVLVMAAVIISGAGGERVPPAAKEPWPPAPHAVVVQQARLEECHRVAPACYTSIGQALAEAGKLVPKVKHRFVVLIKTGE 90
B73  MAAVGRSSLSLVLVMAAVIISGAGSERVPPAAKGALAAGSACCGRTAGAAGGVPPSGSGVLHQHRTSSG--------------------- 69
     ************************ ********

511L YIEQVNITRRNVVLLGEGRGNTVISGNLSNLTGTAMVMTATVNVVADGFLAQNLTIRNEAGPKGRQAVALRSNSNRTVVFGCAIEGFEDS 180
B73  ------------------------------------------------------------------------------------------ 69

511L LYAENGVQVYLETDIYGTVDFIFGNAKAVFQRCRILVRRPIPGKHNVVTAQGCDNNMYENSGFVFHRCSVEADPNPWPVGQNLTGVETFL 270
B73  ------------------------------------------------------------------------------------------ 69

511L GRPYRKYSHVIFMECQLSDVVSAAGWVAWDRAHVINDTTKSVRYMEYANTGPGADTTHRVDWEGVHVLHDPAQVAKYTIDAFISGKEWIP 360
B73  ------------------------------------------------------------------------------------------ 69

511L HQIPYDHEVPSGRGAIIVN 379
B73  ------------------- 69
```

Supplementary Fig. 8 Alignment of *ZmGa2F* predicted protein sequence between 511L and B73.

* indicates the identical amino acids.

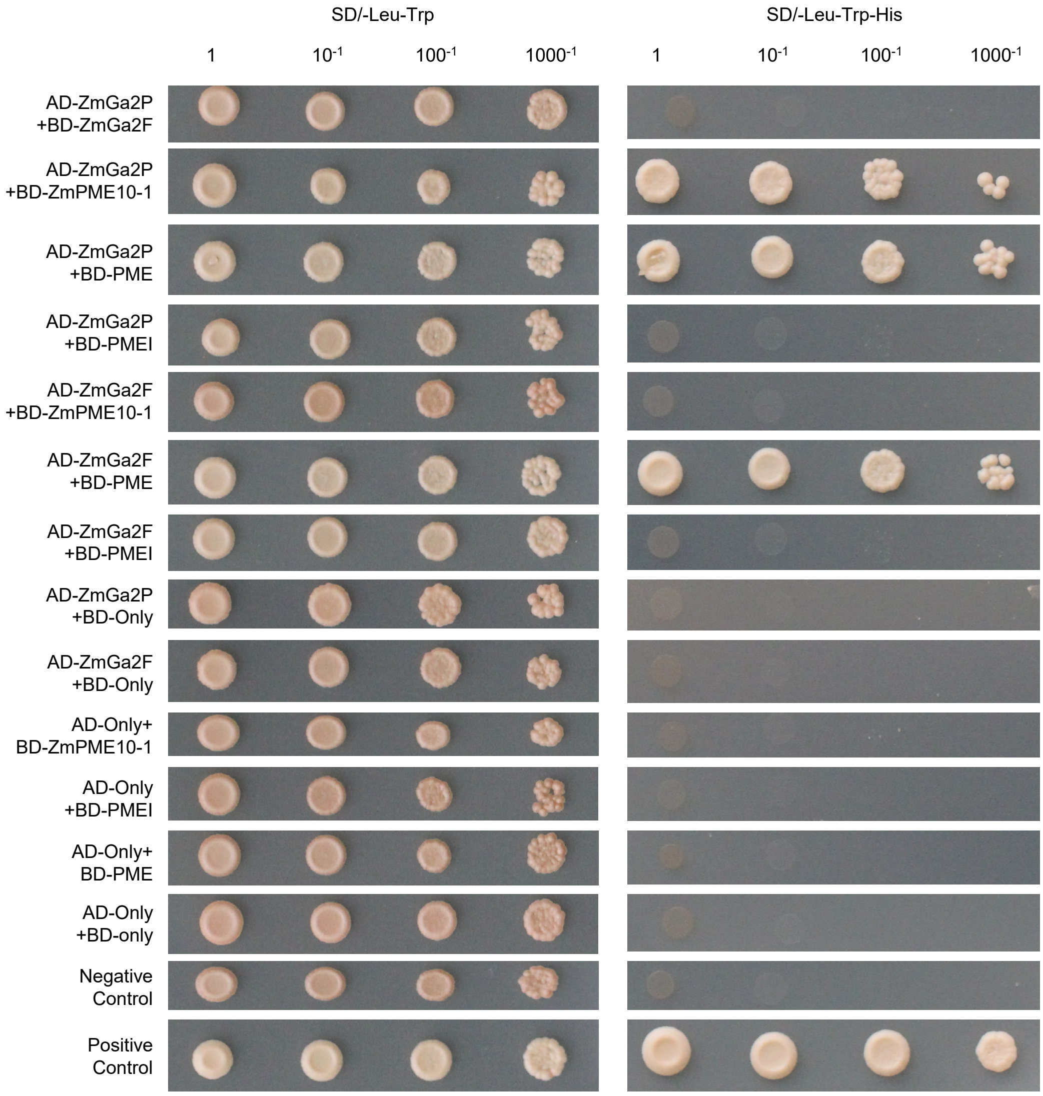

Supplementary Fig. 9 Interactions of ZmGa2P, ZmGa2F and ZmPME10-1 in yeast.

No direct interaction between ZmGa2F and ZmGa2P was detected in yeast. ZmGa2P interacted with both the full length and the PME domain of ZmPME10-1, while ZmGa2F only interacted with the PME domain of ZmPME10-1.

(SD/-Leu-Trp): yeast cells grown on the medium containing no Trp and Leu. (SD/-Leu-Trp-His): yeast cells grown on the medium containing no Trp, Leu, and His. Source data are provided as a Source Data file.

a

```
ZmGa2F MMAAVGRS----SLSLVLVMAAVIISGAGG----ERVPPAAKEP--------------WPPAPHAVVVQQARLEECH--RVAPACYTSIG 65
ZmPme3 MVGGVRRCGLGLAMAVALLLAALVVVASGGAEMRQKLPAGSGNDDDHAAVLSRLSNVIDPPGSWPPRADAVVAKRCGGVAAPPPCYTSIQ 90
Tcb1-f MVGGVRRCGLGLAMAVALLLAALVVVASGGAETRQKLPAGSGNDDDHAAVLSRLSNVIDPPGSWPPRADAVVAKRCRGVAAPPPCYTSIQ 90
       M   V R          L  AA      GG       P                     PP              C        CYTSI

ZmGa2F QALAEAG--KLVPKVKHRFVVLIKTGEYIEQVNITRRNVVLLGEGRGNTVISGNLSNLTGTAMVMTATVNVVADGFLAQNLTIRNEAGPK 153
ZmPme3 AALKAASAPQEAEEVEDKYVVHVLAGVYDETVNITRRNVMLIGDGVGATVITGNKSNATGVHMDMTATVNALGHGFIAQNLTIRNTAGPD 180
Tcb1-f AAVDHAPAPQEAEEVEDKYVVHVLAGVYDETVNITRRNVMLIGDGVGATVITGNKSNATGVHMDMTATVNALGHGFIAQNLTIRNTAGPE 180
        A   A        V    VV      Y E VNITRRNV L   G G TVI GN SN TG  M MTATVN    GF AQNLTIRN AGP

ZmGa2F GRQAVALRSNSNRTVVFGCAIEGFEDSLYAENGVQVYLETDIYGTVDFIFGNAKAVFQRCRILVRRPIPGKHNVVTAQGCDNNMYENSGF 243
ZmPme3 GRQAVALRSNSNKSVVYCCSIEGHEDTLYVENGIQFYLQTSIWGTVDFVFGNAQAMFQSCALLVRRPPKGKHNVLTAQGCNNASRE-SGF 269
Tcb1-f GRQAVALRSNSNKSVVYWCSIEGHEDTLYVENGIQFYLQTSIWGTVDFVFGNAQAMFQSCALLVRRPPKGKHNVLTAQGCNNASRE-SGF 269
       GRQAVALRSNSN  VV  C IEG ED LY ENG Q YL T I GTVDF FGNA A FQ C  LVRRP  GKHNV TAQGC N   E SGF

ZmGa2F VFHRCSVEADPNPWPVGQNLTGVETFLGRPYRKYSHVIFMECQLSDVVSAAGWVAWDRAHVINDTTKSVRYMEYANTGPGADTTHRVDWE 333
ZmPme3 SFHMCTVEAAP-----GVDLDGVETYLGRPYRNFSHVAFIKSYLSRVVSPNGWVAWNKNKVVDDTTRTILYLEYGNDGAGADTAGRVKWP 354
Tcb1-f SFHMCTVEAAP-----GVDLDGVETYLGRPYRNFSHVAFIKSYLSRVVSPNGWVAWNKNKVVEDTTRTILYLEYGNDGAGADTAGRVKWP 354
        FH C VEA P     G  L GVET LGRPYR  SHV F    LS VVS  GWVAW    V  DTT    Y EY N G GADT  RV W

ZmGa2F GVHVLHDPAQVAKYTIDAFISGKEWIPHQIPYDHEVPSGRGAIIVN 379
ZmPme3 GFRVLNTDDEAIAYTADTFINASKWVPEPIQYVHTLGTAPPPRA-- 398
Tcb1-f GFRVLNTDDEAIAYTADTFINASKWVPEPIQYVHTLGTAPPPRA-- 398
       G  VL        YT D FI    W P  I Y H
```

b

```
ZmGa2P MESRRKNQQKTSYIVISIQLLAALLLLELVSLVPGASCEELPLDFWVSALRGVGAKDVSTADGCTKKNKDTVLCSAQANTVTNFINPT-- 88
ZmGa1P -----------MMMSKQMLVLSLLLVLFELGSLPTTSCKKVFFNLWVTNQP----ANATQDAGCAKK--DDALSSADTIKVWNYIDPASQ 73
                           L  LL L      P  SC       WV               GC KK  D  L SA    V N I P

ZmGa2P -NSEEQGYRTIGESIANIPDDSTKRYILILSGGTVYREKVLVSKSKPFVTIRSDDPINPAIIVWNDTAATLGKDSKPLGVDGSSTMTVES 177
ZmGa1P LRPEDGGYTTISESIANIPEDNAKRYLLILKPGVVFREKLLLGRSKPFITIMSEDPMNPAVIVWNDTATTMGKDGKPLGVDGSSTMAIES 163
          E  GY TI ESIANI  D  KRY LIL  G V REK L   SKPF TI S DP NPA IVWNDTA T GKD KPLGVDGSSTM  ES

ZmGa2P DYFIAYGVVFRNDAAAAAKKKKAEGEAPALRVLGTKATFYNCTIEGGQGALYDQMGLHYFKSCTIRGTIDFIFGSAKSFYEDCTIVSVNN 267
ZmGa1P DYFVAYNVVFKNDAPLPKLGEK-KGEAPALRVMGTKATFYNCTIEGGQGALYDQTGLHYFKACAIKGTIDFIFGSAKSFYEECKIVSV-- 250
       DYF AY VVF NDA       K  GEAPALRV GTKATFYNCTIEGGQGALYDQ GLHYFK C I GTIDFIFGSAKSFYE C IVSV

ZmGa2P MEEIMTLPVAPPQLDIHDNPIKVAPGEGGFSFKTCTITGEGQQIFLGRMGTPSIYSYTQIAKEVVPIIYDKGNIFMPSNMTGRRCATFKC 357
ZmGa1P LKEALVLPLAPPEQDRSRNPIEIAPGKSGLAFKTCTIEGEGEKIYLGRVGTPVIYSYTNIGKEIVGIISDGRDVQTVE--RGYYCATFRC 338
         E   LP APP  D   NPI  APG  G  FKTCTI GEG  I LGR GTP IYSYT I KE V II D           G  CATF C

ZmGa2P YGPGLEKIWHVKLRYAEAIYFLGTDFINGDSWILSIPPTDAETLLSV 404
ZmGa1P YGPGMSPMVTSTLTYVEAIPFLGIHYISGESWIPSLPPAEE------ 379
       YGPG        L Y EAI FLG   I   SWI S PP
```

Supplementary Fig. 10 Alignment of protein sequences among maize UCI-related PMEs.

a alignment of ZmGa2F, ZmPme3 and Tcb1-f/PME38. Blue color indicates three identical amino acids. Pink color indicates two identical amino acids.

b alignment of ZmGa2P and ZmGa1P. Blue color indicates identical amino acids.

	*	* *	*	*
P83218	113_ Q A V A L	135_ Q D T L	157_ D F I F G	223_ L G R P W
ZmPME10-1	520_ Q A V A M	542_ Q D T L	564_ D F I F G	360_ L G R P W
ZmGa2P	203_ E A P A L	225_ Q G A L	247_ D F I F G	313_ L G R M G
ZmGa1P	188_ E A P A L	210_ Q G A L	232_ D F I F G	296_ L G R P Y
ZmGa2F	156_ Q A V A L	178_ E D S L	200_ D F I F G	270_ L G R P L
ZmPme3	183_ Q A V A L	205_ E D T L	227_ D F V F G	291_ L G R P Y
PME38	183_ Q A V A L	205_ E D T L	227_ D F V F G	291_ L G R P Y

Supplementary Fig. 11 Catalytic site analysis of maize UCI-related PMEs.

Sequence alignment of conserved amino acids among maize UCI-related PMEs. Asterisks indicate conserved amino acids in catalytic sites. (Q113, Q135, D136, D157, R225; according to the carrot PME P83218 numbering).

a

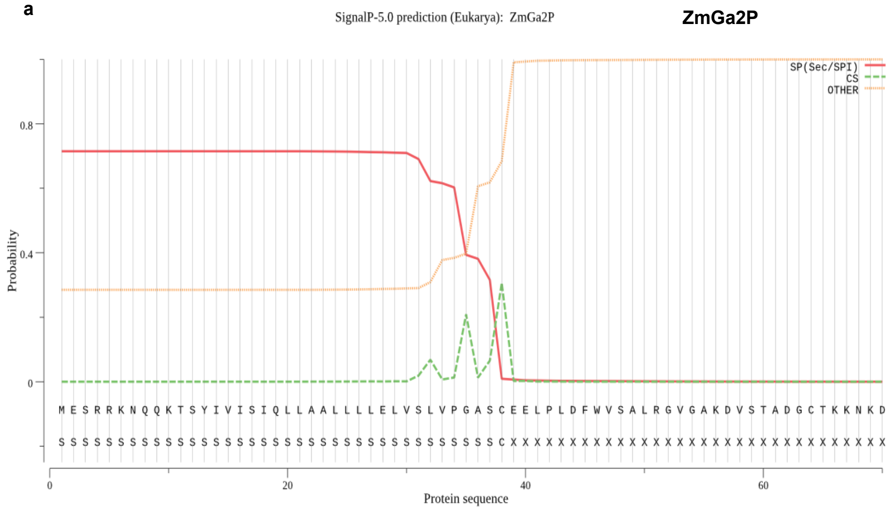

b

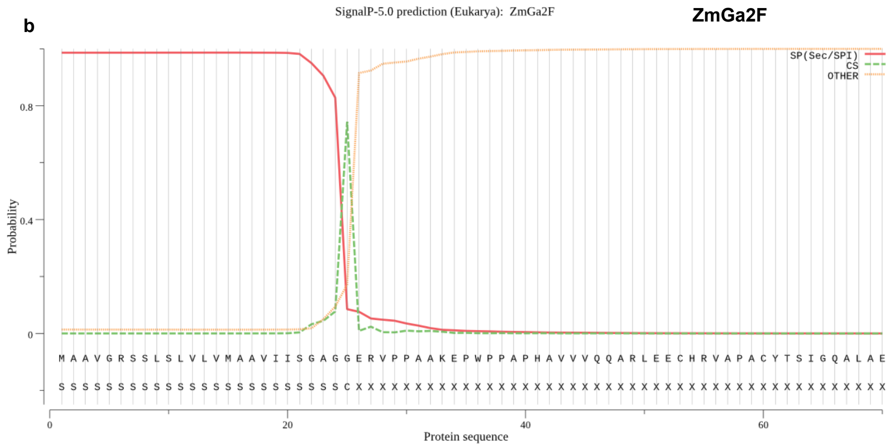

Supplementary Fig. 12 Signal peptide prediction of maize UCI-related PMEs.

Three marginal probabilities: SP(Sec/SPI) (secretory pathway/signal peptide I), CS (the cleavage site) and OTHER (the probability that the sequence does not have any kind of signal peptide). The signal peptide cleavage sites were predicted using the SignalP 5.0 server (https://services.healthtech.dtu.dk/service.php?SignalP-5.0).

a Signal peptide prediction of ZmGa2P.

b Signal peptide prediction of ZmGa2F.

Supplementary Table 1 Most significant DEG of *de novo* assembled transcripts from 511L and B73 silks.

Gene ID	log2 Fold Change	Padj	Position	Merged gene (B73_ RefGenV4)	Annonation
TRINITY_DN1207_c0_g1	13.67341725	8.98E-06	Chr5		Zea mays probable pectinesterase 56
TRINITY_DN644_c0_g1	13.14469639	3.46E-14	Chr4	*Zm00001d026745*	uncharacterized sequence
TRINITY_DN3927_c0_g1	12.5135349	1.23E-12	Chr4		uncharacterized sequence
TRINITY_DN377_c0_g1	12.41975084	2.14E-12	Chr4		uncharacterized sequence
TRINITY_DN1985_c0_g1	12.30214324	3.90E-11	Chr5		uncharacterized sequence
TRINITY_DN5359_c0_g1	12.29487801	4.91E-12	Chr4		uncharacterized sequence
TRINITY_DN390_c0_g4	12.00482821	6.58E-11	Chr6	*Zm00001d035094*	Zea mays 40S ribosomal protein S13
TRINITY_DN5556_c1_g1	11.87813985	5.48E-11	Chr7		uncharacterized sequence
TRINITY_DN10321_c1_g1	11.77890509	1.40E-23	Chr10	*Zm00001d023343*	phospholipid transfer protein homolog1
TRINITY_DN28574_c0_g1	11.7603792	1.04E-10	NA		NA
TRINITY_DN5_c0_g3	11.51007559	1.20E-09	Chr2	*Zm00001d003304*	Bowman-Birk type bran trypsin inhibitor
TRINITY_DN3090_c2_g1	11.34302156	3.94E-09	NA		NA
TRINITY_DN3556_c0_g1	11.31653869	1.35E-09	Chr3		uncharacterized sequence
TRINITY_DN14234_c1_g4	11.2522932	1.72E-09	Chr5	*Zm00001d016565*	Allene oxide cyclase/dirigent protein
TRINITY_DN433_c0_g1	11.19576341	9.05E-10	Chr10		uncharacterized sequence
TRINITY_DN118_c0_g2	11.17549857	2.97E-09	Chr2		uncharacterized sequence
TRINITY_DN563_c0_g1	11.12308952	8.79E-09	Chr8	*Zm00001d010964*	uncharacterized sequence
TRINITY_DN4947_c0_g2	11.12200839	3.96E-09	Chr5	*Zm00001d014207*	uncharacterized sequence
TRINITY_DN726_c3_g2	11.08114688	4.96E-09	Chr3		uncharacterized sequence
TRINITY_DN11749_c0_g1	10.98899423	8.66E-09	Chr6	*Zm00001d035738*	uncharacterized sequence

Top 20 most significant genes. Chromosome location as determined by top BLAST hit to B73_RefGen_V4. Wald test was used to test whether evidence is sufficient to decide against the null hypothesis that there is no effect of the treatment on the gene and that the observed difference between treatment and control was merely caused by experimental variability. The Wald test is two-sided test. Benjamini-Hochberg (BH) method was used to control multiple testing adjustments.

Supplementary Table 2 Outcrossing rates comparison of Zheng58$^{Ga2\text{-}S}$/Chang7-2$^{Ga2\text{-}S}$ and Zheng58^{ga2}/Chang7-2^{ga2} hybrids.

	Beijing			Sanya		
	Purple	Yellow	Percentage	Purple	Yellow	Percentage
Zheng58$^{Ga2\text{-}S}$/Chang7-2$^{Ga2\text{-}S}$	14	3,024	0.4%	10	4,224	0.2%
Zheng58^{ga2}/Chang7-2^{ga2}	1,511	3,155	32.4%	1,732	3,214	35.0%

Supplementary Table 3 Polymorphic markers used for mapping *ZmGa2P* and *ZmGa2F*.

Primers		Primer sequence (5'-3')	Primer position (bp)
M1	Forward	CCGAGAACTTCACCAGAAGG	5:143,455,613-143,455,632
	Reverse	CCACTACGACAGATGCAACG	5:143,456,034-143,456,053
M10	Forward	TAGGATAAGAGCCCGTGAGC	5:150,300,124-150,300,143
	Reverse	CTCCTTGTTTGGGCCTAATG	5:150,300,269-150,300,288
M3	Forward	ATCGGTCGGACAGTTGAGAC	5:151,835,359-151,835,378
	Reverse	TTCTTGGCTCTTGGTCATCC	5:151,834,860-151,834,879
M4	Forward	CACACAACTACGCCAAGCAC	5:152,038,435-152,038,454
	Reverse	GTGGTGAGCCTCTTCCATTC	5:152,038,529-152,038,548
M5	Forward	GCGTTGTATTTCCCCTGAGA	5:152,244,637-152,244,656
	Reverse	GTTTGGTAACACGGGGTTTG	5:152,245,298-152,245,317
M6	Forward	GGCCGAGTTATTAGCTGCTG	5:152,463,366-152,463,385
	Reverse	CGATGAGTGAGGGAGAGAGC	5:152,463,494-152,463,513
M7	Forward	GGTATAAGGCCTGGGCATCT	5:153,426,946-153,426,965
	Reverse	TACTCCACAGCAGGGAGGTC	5:153,427,153-153,427,172
M8	Forward	CGGTTTCCTTGAGGTTGGTA	5:153,532,559-153,532,578
	Reverse	TCGGCATGACCTATGAACAA	5:153,533,018-153,533,037
M9	Forward	GGAATTCCTTGCATCCGTTA	5:156,314,016-156,314,035
	Reverse	GTGGCTGATTGTGACGCTAA	5:156,314,206-156,314,225
M2	Forward	TCCCTGGTGCATACAACAAA	5:165,307,140-165,307,159
	Reverse	GCAACAGGTGGTTTTCTTCC	5:165,307,286-165,307,305

The primer physical position on chromosome 5 is based on the B73_RefGen_V4 sequence released in maizeGDB.

Supplementary Table 4 Primers used in this study.

Primers		Primer sequence (5'-3')	Experimental purpose
ZmGa2F	Forward	ACCGTGAATGTTGTAGCCGAC	Real-time PCR
	Reverse	TCACCACGTTGTGCTTGCC	
Zm00001d016237	Forward	AGACCACTCCCAGACGAACG	
	Reverse	GATCTCCGCGATGATCTTCTG	
Zm00001d016238	Forward	TTGGTTTGCGGCACAATATG	
	Reverse	ATTTTTCTTGAGGGCAACCATT	
Zm00001d016239	Forward	GATCTATCTAGCAGAGATGGCAGC	
	Reverse	AGAAACAGGATATCGTTGAGGAGG	
Zm00001d016242	Forward	GATCGCTGGAACTGTTGGTTATAT	
	Reverse	TCAATCTTTCAGCCCAATCAA	
Zm00001d016245	Forward	ACGCCTTCCATCTACTCCTAC	
	Reverse	CAGCGTCTACCAGTCATATTACT	
Zm00001d016248	Forward	GGGAAGGCACCAACAATAGC	
	Reverse	TGTTCTGTCACCCAAAGAGTCC	
Zm00001d016252	Forward	GCCCAGCAGATTGCTTCTGT	
	Reverse	GTCATCTCAACATTCGGCATCA	
Zm00001d016253	Forward	CCTATTGTTAGTGGCGTGGAGC	
	Reverse	CTCCCGACCGTGTGTATCATC	
ZmGAPDH	Forward	CTGGTTTCTACCGACTTCCTTG	
	Reverse	CGGCATACACAAGCAGCAAC	
ZmGa2P-511L	Forward	ATGGAGAGCAGGAGGAAG	*ZmGa2P* Cloning
	Reverse	TGATGATCTTTGATGATCTCTGAGG	
ZmGa2F-511L	Forward	ATGGCAGCAGTTGGCAGAT	*ZmGa2S* Cloning
	Reverse	GCCGGCTAGTTGACGATGAT	
ZmGa2P-BAC	Forward	ATGGAGAGCAGGAGGAAG	BAC scanning
	Reverse	TGATGATCTTTGATGATCTCTGAGG	
ZmGa2F-BAC	Forward	ATGGCAGCAGTTGGCAGAT	
	Reverse	GGCAGGAGGGAGCAGATGAAT	
ZmGa2P-T	Forward	CAGGAAACAGCTATGAC	Transgenic identification
	Reverse	GAATAGCCTACTCCTCTGC	
ZmGa2P-genomic	Forward	ATGACGGTTGAGGTTGCTTAG	Transgenic vector construction
	Reverse	GAACTTTGGAAAGTGGTGGATA	
ZmGa2F-genomic	Forward	GCCGTCGCCTACTCACAAG	
	Reverse	CGGAGTGGTAGGGGAGGGTA	
M1	Forward	CCGAGAACTTCACCAGAAGG	Hybrid identification
	Reverse	CCACTACGACAGATGCAACG	
M11	Forward	GTGTGGTGTGGAAATCGTGG	
	Reverse	ATGTAGGGCGTACAACAATAGGT	
M5	Forward	GCGTTGTATTTCCCCTGAGA	Pyramiding of *Ga1-S* and *Ga2-S*
	Reverse	GTTTGGTAACACGGGGTTTG	
M6	Forward	GGCCGAGTTATTAGCTGCTG	
	Reverse	CGATGAGTGAGGGAGAGAGC	
ZmGa1P-Del	Forward	GATGGAAGCAGTACCATGGC	
	Reverse	GAGACATCCCAGGCCCGTAAC	

Continued

Primers		Primer sequence (5'-3')	Experimental purpose
ZmPME10-1-511L	Forward	GACAACGATGGCAACATGC	Y2H vector construction
	Reverse	TTACCCTTTGGTGAATCCCA	
ZmPME10-1-PME	Forward	CAGCAGACCCAGAAGCCTAAC	
	Reverse	TTACCCTTTGGTGAATCCCA	
ZmPME10-1-PMEI	Forward	GACAACGATGGCAACATGC	
	Reverse	TTAGATAGCGAGCGCGTTGCTG	
ZmGa2P-511L	Forward	ATGGAGAGCAGGAGGAAG	
	Reverse	TGATGATCTTTGATGATCTCTGAGG	
ZmGa2F-511L	Forward	ATGGCAGCAGTTGGCAGAT	
	Reverse	GCCGGCTAGTTGACGATGAT	
