# Supplementary material for: A pair of non-Mendelian genes at the Ga2 locus confer unilateral cross-incompatibility in maize
Source: Nat Commun. 2022 Apr 14;13:1993. doi: 10.1038/s41467-022-29729-z (PMC9010485; doi:10.1038/s41467-022-29729-z)
Supplement: Supplementary file 3 — Description of Additional Supplementary Files [file 41467_2022_29729_MOESM3_ESM.pdf]

Description of Additional Supplementary Files

File name: Supplementary Data 1

Description: Haplotypes of the 946 inbred lines at *Ga2* locus.

File Name: Supplementary Data 2

Description: The BAC sequence of 511L (*Ga2-S*) containing *ZmGa2P*.

File Name: Supplementary Data 3

Description: The sequences of top 20 most significant DEG of *de novo* assembled transcripts.

File Name: Supplementary Data 4

Description: The BAC sequence of 511L (*Ga2-S*) containing *ZmGa2F*.
