# Supplementary material for: A pair of non-Mendelian genes at the Ga2 locus confer unilateral cross-incompatibility in maize
Source: Nat Commun. 2022 Apr 14;13:1993. doi: 10.1038/s41467-022-29729-z (PMC9010485; doi:10.1038/s41467-022-29729-z)
Supplement: Supplementary file 8 — Reporting Summary [file 41467_2022_29729_MOESM8_ESM.pdf]

nature portfolio

Corresponding author(s): Huabang Chen, Juan Liu

Last updated by author(s): Mar 17, 2022

Reporting Summary

Nature Portfolio wishes to improve the reproducibility of the work that we publish. This form provides structure for consistency and transparency in reporting. For further information on Nature Portfolio policies, see our Editorial Policies and the Editorial Policy Checklist.

Statistics

For all statistical analyses, confirm that the following items are present in the figure legend, table legend, main text, or Methods section.

n/a	Confirmed	
☐	☒	The exact sample size (*n*) for each experimental group/condition, given as a discrete number and unit of measurement
☐	☒	A statement on whether measurements were taken from distinct samples or whether the same sample was measured repeatedly
☐	☒	The statistical test(s) used AND whether they are one- or two-sided<br>*Only common tests should be described solely by name; describe more complex techniques in the Methods section.*
☒	☐	A description of all covariates tested
☐	☒	A description of any assumptions or corrections, such as tests of normality and adjustment for multiple comparisons
☐	☒	A full description of the statistical parameters including central tendency (e.g. means) or other basic estimates (e.g. regression coefficient) AND variation (e.g. standard deviation) or associated estimates of uncertainty (e.g. confidence intervals)
☐	☒	For null hypothesis testing, the test statistic (e.g. *F*, *t*, *r*) with confidence intervals, effect sizes, degrees of freedom and *P* value noted<br>*Give P values as exact values whenever suitable.*
☒	☐	For Bayesian analysis, information on the choice of priors and Markov chain Monte Carlo settings
☒	☐	For hierarchical and complex designs, identification of the appropriate level for tests and full reporting of outcomes
☐	☒	Estimates of effect sizes (e.g. Cohen's *d*, Pearson's *r*), indicating how they were calculated

Our web collection on statistics for biologists contains articles on many of the points above.

Software and code

Policy information about availability of computer code

Data collection	RNA-seq data of silks of 511L and B73 (Int. J. Mol. Sci., 2018, 19, 1757) were used in this study. No software for data collection was used.
Data analysis	The quality control of raw data was processed by Fastp software (Bioinformatics, 2018, 34, i884-i890), which cut the front 15-bp of each read from 5' to 3' and removed low-quality reads and adapters. Trinity software (Nat. Biotechnol., 2011, 29, 644-652) was used to de novo assemble the transcriptome, and the default parameters were used in the command line. The transcripts abundances were computed by Salmon software (Nat. Methods, 2017, 14, 417-419). The DEGs between samples were identified by DESeq2 package (Genome Biol., 2014, 15, 1-21) in R.

For manuscripts utilizing custom algorithms or software that are central to the research but not yet described in published literature, software must be made available to editors and reviewers. We strongly encourage code deposition in a community repository (e.g. GitHub). See the Nature Portfolio guidelines for submitting code & software for further information.

Data

Policy information about availability of data

All manuscripts must include a data availability statement. This statement should provide the following information, where applicable:

- Accession codes, unique identifiers, or web links for publicly available datasets
- A description of any restrictions on data availability
- For clinical datasets or third party data, please ensure that the statement adheres to our policy

Data supporting the findings of this work are available within the manuscript and its Supplementary Information files. The RNA-seq datasets are available from the National Center for Biotechnology Information, the BioProject and SRA accession numbers are (https://www.ncbi.nlm.nih.gov/bioproject/PRJNA808835/) and SRR18086820-SRR18086823. Source data are provided with this paper.

Field-specific reporting

Please select the one below that is the best fit for your research. If you are not sure, read the appropriate sections before making your selection.

- [x] Life sciences
- [ ] Behavioural & social sciences
- [ ] Ecological, evolutionary & environmental sciences

For a reference copy of the document with all sections, see nature.com/documents/nr-reporting-summary-flat.pdf

Life sciences study design

All studies must disclose on these points even when the disclosure is negative.

Sample size	Required experimental sample sizes were estimated based on our past experience performing similar experiments.
Data exclusions	No data were excluded from our analyses.
Replication	All experiments were repeated at least three times.
Randomization	For all experiments involving phenotyping or tissue collection from plants, segregating populations and a randomized block design were used. The genotypes were planted in a randomly distribution in the field. This ensured that all genotypes were randomized within each experiment.
Blinding	Analysis were performed in a manner blinded to treatments in all experiments.

Reporting for specific materials, systems and methods

We require information from authors about some types of materials, experimental systems and methods used in many studies. Here, indicate whether each material, system or method listed is relevant to your study. If you are not sure if a list item applies to your research, read the appropriate section before selecting a response.

Materials & experimental systems

n/a	Involved in the study	
[ ]	[x]	Antibodies
[x]	[ ]	Eukaryotic cell lines
[x]	[ ]	Palaeontology and archaeology
[x]	[ ]	Animals and other organisms
[x]	[ ]	Human research participants
[x]	[ ]	Clinical data
[x]	[ ]	Dual use research of concern

Methods

n/a	Involved in the study	
[x]	[ ]	ChIP-seq
[x]	[ ]	Flow cytometry
[x]	[ ]	MRI-based neuroimaging

Antibodies

Antibodies used	LM20, an antibody against highly esterified homogalacturonan (#AB-LM20, Megazyme) at a 1:20 dilution was used in Immunolabeling analysis.
Validation	Verhertbruggen, Y., Marcus, S.E., Haeger, A., Ordaz-Ortiz, J.J. & Knox, J.P. An extended set of monoclonal antibodies to pectic homogalacturonan. Carbohydr. Res. 344, 1858-1862 (2009).
